# *ErbB3 *is required for ductal morphogenesis in the mouse mammary gland

**DOI:** 10.1186/bcr2198

**Published:** 2008-11-18

**Authors:** Amy J Jackson-Fisher, Gary Bellinger, Jerrica L Breindel, Fatteneh A Tavassoli, Carmen J Booth, James K Duong, David F Stern

**Affiliations:** 1 Department of Pathology, Yale School of Medicine, New Haven, CT 06520-8023, USA; 2 Current address: Pfizer, Science Center Drive, San Diego, CA 92121, USA; 3 Current address: Pfizer, Eastern Point Road, Groton, CT 06340, USA; 4 Section of Comparative Medicine, Yale School of Medicine, New Haven, CT 06520-8023, USA; 5 School of Public Health, Columbia University, New York, NY 10032, USA

## Abstract

**Introduction:**

The receptor *ErbB3*/*HER3 *is often over-expressed in human breast cancers, frequently in conjunction with over-expression of the proto-oncogene *ERBB2*/*HER2*/*NEU*. Although the prognostic/predictive value of ErbB3 expression in breast cancer is unclear, ErbB3 is known to contribute to therapeutic resistance. Understanding ErbB3 functions in the normal mammary gland will help to explain its role in cancer etiology and as a modulator of signaling responses to the mammary oncogene *ERBB2*.

**Methods:**

To investigate the roles of ErbB3 in mouse mammary gland development, we transplanted mammary buds from *ErbB3*^-/- ^embryos into the cleared mammary fat pads of wild-type immunocompromised mice. Effects on ductal outgrowth were analyzed at 4 weeks, 7 weeks and 20 weeks after transplantation for total ductal outgrowth, branch density, and number and area of terminal end buds. Sections of glands containing terminal end buds were analyzed for number and epithelial area of terminal end buds. Terminal end buds were also analyzed for presence of mitotic figures, apoptotic figures, BrdU incorporation, and expression of E-cadherin, P-cadherin, α-smooth muscle actin, and cleaved caspase-3.

**Results:**

The mammary ductal trees developed from *ErbB3*^-/- ^buds only partly filled the mammary fat pad. In contrast to similar experiments with *ErbB2*^-/- ^mammary buds, this phenotype was maintained through adulthood, pregnancy, and parturition. In addition, and in contrast to similar work with *ErbB4*^-/- ^mammary buds, lobuloalveolar development of *ErbB3*^-/- ^transplanted glands was normal. The *ErbB3*^-/- ^mammary outgrowth defect was associated with a decrease in the size of the terminal end buds, and with increases in branch density, in the number of terminal end buds, and in the number of luminal spaces. Proliferation rates were not affected by the lack of *ErbB3*, but there was an increase in apoptosis in *ErbB3*^-/- ^terminal end buds.

**Conclusions:**

Endogenous ErbB3 regulates morphogenesis of mammary epithelium.

## Introduction

The epidermal growth factor receptor (EGFR) family of receptor tyrosine kinases, which consists of EGFR, ErbB2, ErbB3, and ErbB4, is important in many normal developmental processes and is often over-expressed or mutated in human cancer (for review, see [1]). Ligand binding stimulates homodimerization and heterodimerization of EGFR family members. The dimerization facilitates cross-phosphorylation of tyrosines within the cytoplasmic domains that become binding sites for downstream effector proteins. The proto-oncogene product ErbB2 is unique in that it does not bind soluble ligands and requires a heterodimer partner for ligand-dependent activation. ErbB3 is also unique because the kinase domain is catalytically inactive, so that ErbB3 also requires a heterodimerization partner for activity. Once activated, ErbB3 is strongly linked to prosurvival signaling through the phosphatidylinositol 3'-kinase (PI3K)/Akt pathway. ErbB3 has six binding sites for the p85 adaptor subunit of PI3K. PI3K catalyzes formation of 3' phosphoinositides that recruit the protein kinase Akt/protein kinase B to the membrane for activation by phosphorylation. The activated Akt is involved in cellular processes including survival, cell growth, and proliferation (for review, see [2]).

The ErbB2/ErbB3 heterodimer, one of many possible ErbB heterodimer combinations, is important in tumorigenesis [3-6] (for review, see [7]). ErbB3 is a preferred heterodimerization partner for the proto-oncogene ErbB2, and the ErbB2/ErbB3 heterodimer is highly biologically active and pro-tumorigenic *in vitro *[8]. ErbB2 and ErbB3 are often co-over-expressed in breast, ovarian, colorectal, and bladder cancers [9-13].

Recent advances in the treatment of cancers driven by ErbB alterations include the use of small molecule tyrosine kinase inhibitors and antagonist antibodies [14]. Surprisingly, ErbB3 signaling is often crucial to the success or failure of ErbB tyrosine kinase inhibitors, despite the lack of intrinsic ErbB3 kinase activity. In breast cancers with amplified *ERBB2*, ErbB3 augments ErbB2 signaling through strong coupling to survival pathways that complement the signals emanating from ErbB2, thus allowing the cancer cells to escape from inhibition therapy [15]. In all likelihood, ErbB3 signaling will also contribute to resistance to single and dual specificity EGFR and ErbB2 inhibitors (for example, lapatinib) [16], as they come into clinical use for trastuzumab-resistant *ERBB2*-amplified breast cancer, and to pertuzumab, an antibody inhibitor of ErbB2/ErbB3 heterodimerization [17]. In non-small-cell lung carcinoma (NSCLC) cells with mutated EGFR, amplification of the *MET *oncogene activates prosurvival ErbB3 signaling, which allows escape from tyrosine kinase inhibition [18] (for review, see [19]). Given these findings, downregulation of ErbB3 signaling in human cancers is critical to successful ErbB-targeted tyrosine kinase inhibitor therapy.

The importance of ErbBs in human breast cancer is linked to their importance in normal mammary gland biology. Understanding these normal functions and their regulators will lead to better prognostication and treatment decisions, and to identification of novel therapeutic targets. However, the individual role played by each receptor in mammary gland development has been difficult to determine. This is because the individual receptors and their agonists display complex and overlapping expression patterns, and because the receptors are required for multiple developmental activities, some of which are essential for prenatal viability [20,21]. Both EGFR and ErbB2 are highly expressed and co-localized in all major cell types in the pubescent mouse mammary gland, but at maturity they are differentially localized, with EGFR in the stroma and ErbB2 in the epithelium [20]. ErbB3 and ErbB4 are only expressed at low levels in postpubescent mammary glands from virgin mice, but they are expressed at higher levels during pregnancy and lactation [20,22]. All four of the ErbBs are expressed in the late pregnancy and early lactation glands. This differential expression pattern implies important roles for EGFR and ErbB2 in the developing mammary gland, and for ErbB3 and ErbB4 in the later stages of mammary gland development and differentiation.

Early studies suggested that only ErbBs EGFR and ErbB2 are important in early postnatal mammary development, because ErbB3 and ErbB4 are expressed at very low levels, and because neuregulins (NRGs), growth factors that bind exclusively to ErbB3 and ErbB4, were difficult to detect at early stages [20]. Nonetheless, there is evidence for ErbB3 or ErbB4 signaling in the pubescent mammary gland. Implantation of Elvax ethylene vinyl acetate (Elvax Ethylene, Dupont, Wilmington, Delaware, USA) slow release pellets containing NRG1, normally expressed during pregnancy, promoted ductal growth and alveologenesis in mammary glands of pubescent mice [23]. Because NRG1 binds ErbB3 and ErbB4 but not EGFR or ErbB2, this suggested that ErbB3 and/or ErbB4 are functional (but not necessarily active) at puberty. *ErbB4 *null mice, with the essential cardiac function supplied by transgene-driven expression of ErbB4 cDNA exclusively in the heart, survive past birth and are defective in lactation, but undergo normal development at puberty [24]. This indicates that ErbB4 is not essential during pubescent mammary gland development, and implies, by elimination, that ErbB3 is the active receptor in NRG1 implantation experiments.

Attempts to determine the role of ErbB3 in mammary gland development have been impeded by the embryonic lethality caused by *ErbB3 *gene disruption, which leads to cardiac and neurodevelopmental defects [25]. To determine the roles played by ErbB3 in mammary gland development, we chose to use a loss-of-function approach. Mammary buds isolated from *ErbB3 *null embryos at day 12.5 were transplanted into the cleared fat pad of prepubescent immunocompromised recipient mice. Transplanted *ErbB3 *null buds supported growth of an epithelial tree, but the ducts did not penetrate the fat pad to the same extent as ducts from a wild-type bud transplant. Also, the terminal end buds (TEBs) that pilot ductal extension through the mammary fat pad have an increase in apoptotic structures.

## Materials and methods

### Mammary gland transplants

A null mutation was created at the *ErbB3 *gene locus by homologous recombination resulting in the deletion of amino acids 73 to 107. This results in embryonic lethality as early as day 13.5 [25]. Embryonic day 12.5 embryos (stage estimated from timed pregnancies) resulting from an intercross of *ErbB3*^+/- ^heterozygotes in a mixed 129/C57BL6/BalbC or a high C57BL6 strain background were harvested by Caesarean section. Immunoblot analysis of *ErbB3*^+/+^, *ErbB3*^+/-^, and *ErbB3*^-/- ^embryos verified dose-dependent expression of ErbB3 (Additional data file 1). Mammary gland transplants were performed as described in [26]. For each recipient mouse, one number four inguinal mammary fat pad was transplanted with a single mammary bud isolated from a *ErbB3*^-/- ^female, and the contralateral number four inguinal fat pad was transplanted with a mammary bud from an *ErbB3*^+/+ ^or *ErbB3*^+/- ^female littermate. The fat pads containing transplants were harvested from virgin hosts at 4, 7, or 20 weeks after transplantation, or the recipients were bred and the glands harvested at 1 day postpartum. The overall take rate for transplanted buds was 74% and was not affected by the genotype of the donor embryo. All animal work was approved by the Yale University Institutional Animal Care and Use Committee and followed internationally recognized guidelines.

### Morphological analysis

Whole mount analysis was carried out as described in [23,26]. Four +/+ and four -/- gland whole mounts of outgrowths were analyzed at 4 weeks after transplantation; three +/+, six +/- and five -/- glands 7 weeks after transplantation; one +/+, one +/- and two -/- glands at 20 weeks after transplantation; four +/- and five -/- glands at 1 day postpartum, after the first pregnancy; and one +/+ and one -/- at 1 day postpartum, after the second pregnancy.

Histological analysis was performed as described in [26,27]. Five +/+, three +/-, and seven -/- gland outgrowths were analyzed 4 weeks after transplant; three +/+ or +/- and three -/- glands at 7 weeks after transplant; one +/+ and two -/- glands at 20 weeks after transplantation; three +/- and four -/- glands at 1 day postpartum, after the first pregnancy; and two +/+ or +/- and two -/- 1 day postpartum, after the second pregnancy.

Measurements of total ductal outgrowth length on whole glands (no half glands) and branch density (whole and half glands) were taken as described in [26], except with the use of ImageJ imaging software (Wayne Rasband, National Institutes of Health, Bethesda, Maryland, USA). Briefly, the total ductal outgrowth in digital images of glands was determined by drawing a line from the one edge of furthest ductal outgrowth to the opposite edge of furthest ductal outgrowth, and measuring the length of that line in arbitrary units. This analysis was done in triplicate for each gland and the average measurement is reported. The branchpoint analysis was done by counting the number of branchpoints within a box of fixed dimensions. The area of TEBs in digital images of carmine alum-stained whole mounted glands was determined by outlining the TEB using the elliptical or freehand selection tool in ImageJ imaging software. The epithelial area of the TEBs in the sections from paraffin-embedded glands were analyzed similarly, but areas of luminal and other vacuolated spaces were subtracted from the outer measurements to give the epithelial area only.

### Immunohistochemistry

Immunohistochemistry (IHC) for E-cadherin, P-cadherin, α-smooth muscle actin (SMA) were performed as described by Jackson-Fisher and coworkers [26], except a 1:200 dilution of primary antibody was used for SMA IHC. For E-cadherin IHC, seven +/+, four +/-, and 10 -/- glands were analyzed; and for SMA IHC six +/+, four +/-, and 10 -/- glands were analyzed. Mouse IgG was used as a negative control for both E-cadherin and SMA IHC. For P-cadherin IHC, seven +/+, four +/-, and 10 -/- glands were analyzed and goat IgG was used as a negative control.

### BrdU and cleaved caspase 3

5-Bromo-2-deoxyuridine (BrdU) analysis (n = 3 +/+, 1 +/-, and 6 -/-) was performed as described previously [26,28]. For cleaved caspase 3 IHC (n = 5 +/+, 3 +/-, and 7 -/- glands), rabbit polyclonal cleaved caspase-3 (Asp175) antibody (Cell Signaling Technology, Danvers, MA, USA) was used. The antigen was unmasked in a low pH citrate buffer under heat and pressure. The antibody was diluted 1:100 in Primary Antibody Diluting Buffer (Biomeda, Foster City, CA, USA), overnight at 4°C. Endogenous peroxidases were inactivated with 1% hydrogen peroxide 10 minutes, and a 1:100 diluted biotinylated anti-rabbit secondary antibody solution (Vectastain Elite ABC kit, PK-6101; Vector Laboratories, Burlingame, CA, USA) was added. Peroxidase activity was detected with freshly prepared DAB solution (Biogenex, San Ramon, CA, USA).

### Histological analysis of apoptosis

In a double-blind analysis, five TEBs on each hematoxylin and eosin (H&E) stained section were scored for total number of nuclei and for the number of apoptotic cells (n = 6 +/+ or +/-, and 7 -/- glands; 25 slides total). Slides were examined using an Axioskop microscope (Carl Zeiss Microimaging, Thornwood, NJ, USA). Mammary bud epithelial cell nuclei and apoptotic cells within a 100 square (1 mm × 1 mm) grid were counted manually on a laboratory counter (Denominator, Woodbury, CT, USA). Five separate mammary buds were counted for one tissue section per slide at 40×. The ratio of apoptotic cells to nuclei was determined.

## Results

Analysis of *ErbB3 *function in postnatal development of the mammary gland has been hampered by the embryonic lethality of *ErbB3 *knockout mice. *ErbB3*^-/- ^null mice die *in utero *at day 13.5 or as late as days 16 to 18, depending on the strain background, because of cardiac cushion abnormalities [25]. However, postnatal development of *ErbB3*^-/- ^epithelium in ErbB3 wild-type stroma can be analyzed by transplantation. Mammary buds from day 12.5 *ErbB3*^-/- ^embryos were isolated and transplanted into prepubescent immunocompromised recipient mammary fat pads that had been surgically cleared of the endogenous epithelium. The contralateral gland of each recipient mouse was also cleared of epithelium and transplanted with an *ErbB3 *wild type (+/+) or heterozygous (+/-) mammary bud from a female littermate embryo as a positive control. At various times after transplantation, the transplanted glands were harvested from the recipient mice and the epithelial tree was visualized by carmine alum staining of whole mounted glands.

**Figure 1:**
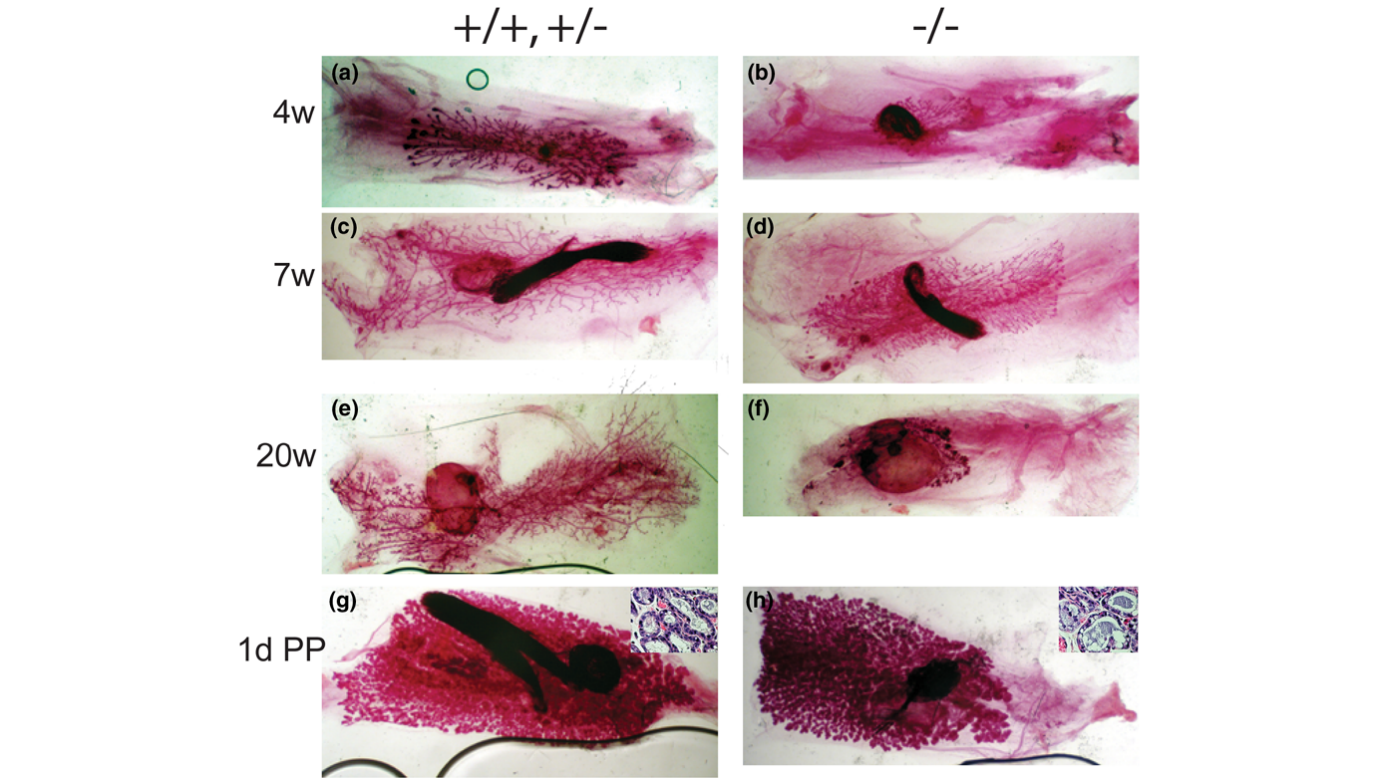
Whole mount analysis of ductal morphogenesis in transplanted glands. Mammary buds from *ErbB3*^+/+ ^or *ErbB3*^+/- ^and *ErbB3*^-/- ^day 12.5 embryos were transplanted into contralateral cleared mammary fat pads of 3-week-old *Rag1*^-/- ^females. The glands were harvested at **(a, b) **4 weeks, **(c, d) **7 weeks, **(e, f) **20 weeks after transplantation, or **(g, h) **1 day postpartum. Shown are paired contralateral transplanted glands from the same recipient mouse. The 20-week glands were from a mouse that was mated but did not deliver a litter and was not pregnant at time of sacrifice. The 1 day postpartum (1D PP) mouse was mated about 8 weeks after transplantation and a litter was delivered about 12 weeks after transplantation. The glands were harvested 1D PP. Inserts in panels g and h are hematoxylin and eosin stained sections of +/+ (panel g) and -/- (panel h) paired transplants at 1D PP.

**Figure 2:**
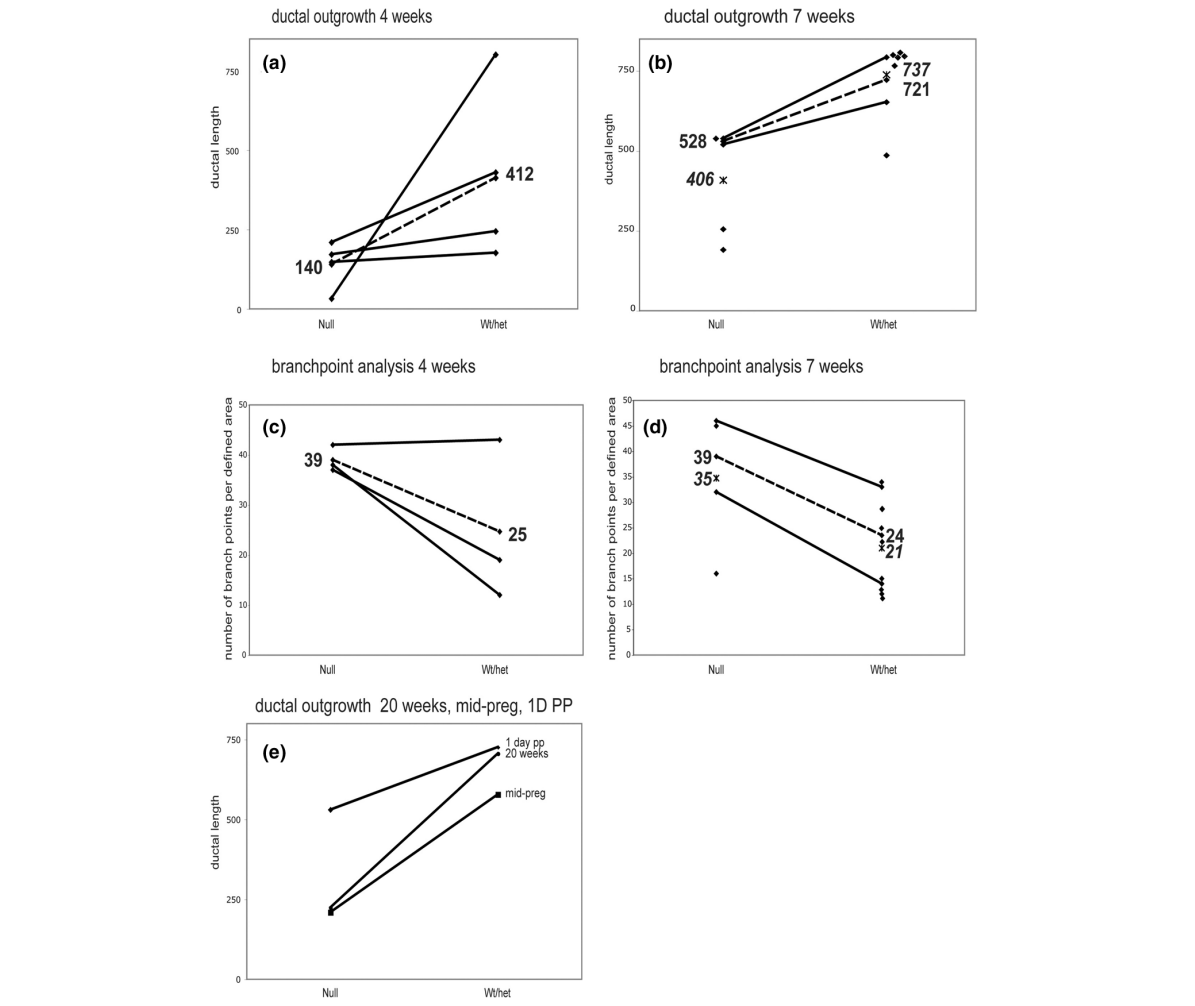
Analysis of epithelial outgrowth. Diamonds represent the average end to end length of total ductal outgrowth measured in arbitrary units in a transplanted gland at **(a) **4 weeks and **(b) **7 weeks for the null and wild-type or heterozygous (Wt/het) donor genotypes. The average numbers of branchpoints within a unit area are shown for **(c) **4 weeks and **(d) **7 weeks after transplantation. **(e)** Ductal outgrowths for 20 weeks after transplantation, mid-pregnancy, and 1 day postpartum (1D PP) are shown together. In all panels, solid lines connect the null with the Wt/het pair of contralateral transplants from the same recipient mouse. Dotted lines connect the mean of the outgrowths from contralateral glands in complete null-Wt/het pairs, and the mean lengths are listed at the termini of these lines. Asterisks represent the mean from all recipients of a given genotype, with mean lengths presented in italics in panels b and d.

At 4 weeks after transplantation the epithelial trees resulting from *ErbB3*^+ ^(+/+ or +/-) transplant had filled a majority of the fat pad (Figures 1a and 2a), whereas the *ErbB*^-/- ^transplant epithelial trees only filled a small fraction of the glands (Figures 1b and 2a). The total ductal length was measured for each of the paired contralateral gland outgrowths (four pairs). The length of the *ErbB3*^+ ^outgrowths and the severity of the *ErbB3*^-/- ^penetration defect varied at 4 weeks (Figure 2a). The mean *ErbB3*^+ ^outgrowth length was 412; the mean *ErbB3*^-/- ^outgrowth was 140. At seven weeks after transplantation, most of the *ErbB3*+ outgrowths (eight glands) had completely filled the fat pad (Figures 1c and 2b), but none of the *ErbB3*^-/- ^outgrowths (five glands) filled the fat pad (Figures 1d and 2b). The severity of the *ErbB3*^-/- ^ductal penetration defects varied. At 7 weeks, transplantation yielded two 'paired' glands from mice in which both *ErbB3*^+ ^and *ErbB3*^-/- ^transplants were successful. For these pairs only, the mean *ErbB3*^+ ^outgrowth length was 721; the mean *ErbB3*^-/- ^outgrowth was 528 (Figure 2b). For all of the glands assayed, including a number of 'unpaired' glands from mice in which only one of the two transplants survived, the mean *ErbB3*^+ ^outgrowth length was 737; the mean *ErbB3*^-/- ^outgrowth was 406. This ductal penetration defect persisted in adult nonparous *ErbB3*^-/- ^glands (two glands; Figures 1f and 2e), and was not rescued by the influence of hormones during pregnancy or lactation (Figure 1h and 2e). *ErbB3*^+ ^outgrowths (four glands) filled the fat pad and normal alveolar structures were evident at 1 day postpartum (Figures 1g and 2e). Even though the *ErbB3*^-/- ^outgrowth did not reach the edges of the fat pad, normal alveolar structures were present at 1 day postpartum (Figures 1h and 2e; four *ErbB3*^+ ^glands and four *ErbB3*^-/- ^glands). On the histological level, the alveoli were distended, with milk in the lumens and lipid droplets in the cells, suggesting that both the differentiation and secretory pathways are normal in the *+/+ *and *-/- *glands (insets, Figure 1g,h). Analysis of lactation after 1 day postpartum is not possible because the epithelial outgrowth from the transplant is not attached to the nipple, so remodeling of the gland to the prepregnancy state occurs quite rapidly.

**Figure 3:**
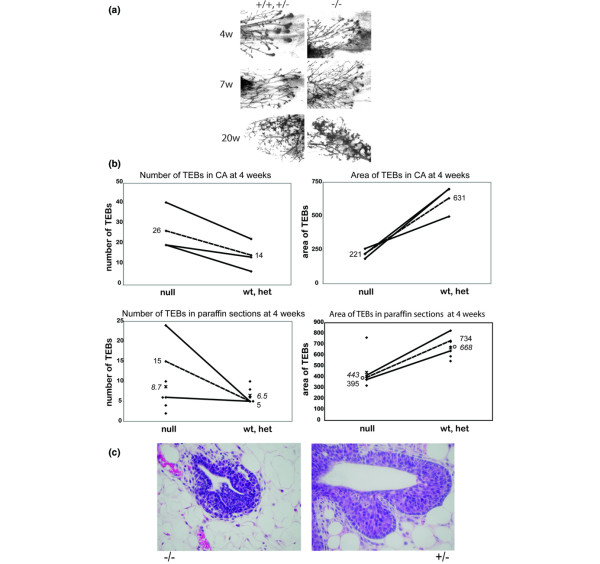
Whole mount analysis of TEBs. **(a) **High-magnification TEBs in carmine alum stained whole mounts. Large bulbous TEBs are present at the end of the advancing duct in +/+,+/- glands at 4 weeks after transplantation (top left), but have regressed by 7 weeks (middle left) and 20 weeks (bottom left) after transplantation. The TEBs in the -/- glands are present at 4 weeks (top right), they persist at 7 weeks (middle right), and have regressed by 20 weeks (bottom right). Whole mounts at a given time point are contralateral glands from the same recipient mouse at the same magnification. **(b) **Number and area of TEBs after transplantation. Shown are the number of TEBs in a carmine alum (CA)-stained whole mount (top left) and the average epithelial area of the TEBs (top right) at 4 weeks after transplantation in three mice with paired contralateral outgrowth; and the number of TEBs (bottom left) and the epithelial area of the TEB in stained paraffin-embedded sections (bottom right) at 4 weeks aftertransplantation in six *ErbB3*^-/- ^glands and six *ErbB3*^+ ^glands, two of which are paired contralateral outgrowths. Solid lines connect the paired null and wild-type or heterozygous (wt/het) contralateral transplants from the same recipient mouse. Dotted lines connect the mean of the outgrowths from contralateral glands in the complete null-wt/het pairs, and the means are listed at the termini of these lines. Asterisks represent the mean from all recipients of a given genotype, with means listed in italics. Circles mark the TEBs shown in panel c. **(c) **Representative TEBs in paraffin-embedded sections. An *ErbB3*^+ ^TEB (bottom right) in an hematoxylin and eosin stained paraffin-embedded section compared with a *ErbB3*^-/- ^TEB (bottom left) taken at the same magnification. Both TEBs were near average size for the respective genotype. Circles in panel b, lower right, mark the corresponding diamonds. TEB, terminal end bud.

In addition to the ductal penetration defect, there was an increase in the number of branchpoints in the *ErbB3*^-/- ^outgrowths in comparison to the *ErbB*^+ ^outgrowths (Figure 2c,d). Likewise, there was an increase in the number of TEBs in *ErbB3*^-/- ^glands (Figure 3a). TEBs are bulbous structures found at the invasive ends of an advancing ducts, and are major sites of proliferation and apoptosis in the pubescent mammary gland [29]. Normally, TEBs regress once the developing epithelial tree has reached the edge of the fat pad. At 4 weeks after transplantation, several large TEBs are evident in whole mounts of *ErbB3*^+ ^outgrowths (Figure 3a, top left). however, by 7 weeks after transplantation (Figure 3a, middle left) the TEBs have mainly regressed and they are not present at 20 weeks after transplantation (Figure 3a, bottom left). TEBs are visible in *ErbB3*^-/- ^glands at 4 weeks (Figure 3a, top right) and at 7 weeks (Figure 3a, middle right). Interestingly, they have regressed by 20 weeks after transplantation (Figure 3a, bottom right), despite the incomplete extension through the fat pad. The number of TEBs visible in both carmine alum stained whole mounted glands (Figure 3b, top left) and across a series of sections from paraffin-embedded whole glands (Figure 3b, bottom right) was greater in *ErbB3*^-/- ^glands than in *ErbB3*^+ ^glands. However the epithelial area of the TEBs was smaller for the *ErbB3*^-/- ^glands than for the *ErbB3*+ glands. This was observed in both the carmine alum stained whole mounts (Figure 3b, top right) and in the sections from the paraffin-embedded glands (Figure 3b, bottom right). TEBs from *ErbB3*^+ ^glands were larger in size and epithelial area (Figure 3c, right) than TEBs from *ErbB3*^-/- ^glands (Figure 3c, left); the corresponding glands are marked with circles in Figure 3b (lower right). In the carmine alum analysis (three pairs), the mean area of the *ErbB3*^+ ^TEBs was 631 whereas the mean area of the *ErbB3*^-/- ^TEBs was 221. In an analysis of the paraffin sections, the mean area of the *ErbB3*^+ ^TEBs (six glands) was 668, whereas the mean area of the *ErbB3*^-/- ^TEBs (six glands) was 443. In analysis of only the paired paraffin sections (two pairs), the mean area of the *ErbB3*^+ ^TEBs was 734 whereas the mean area of the *ErbB3*^-/- ^TEBs was 395.

**Figure 4:**
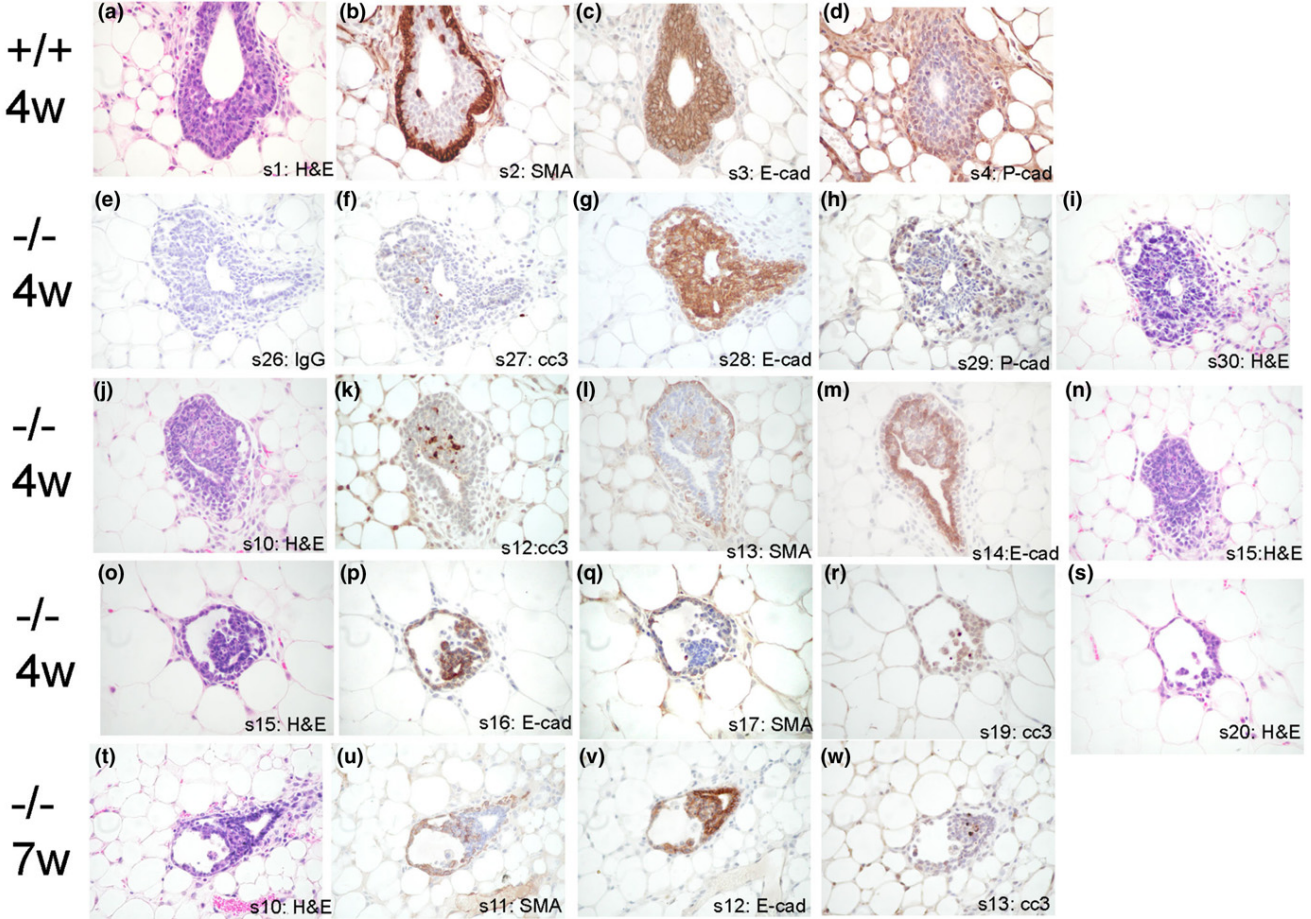
Immunohistochemical analysis of a TEB in serial sections. **(a-d) **TEBs from serial sections of an *ErbB3*^+/+ ^gland 4 weeks after transplantation (panels a-d) have a typical single layer cap cell/myoepithelial cell outer layer (panels b and d) and a multilayered body cell section (panel c). **(e-i, j-n, and o-s) **Serial sections of three representative -/- TEBs at 4 weeks after transplantation. The three TEBs vary in the cap cell layer (panels h, l, and q), in the layers in the body cell layer (panels g, m, and d), and in the size of the spaces between the cap and body cell layers (panels i, j, and o). **(t-w) **The multilayered cap cell compartment has large spaces and the body cell layer is a single layer in the serial section of an *ErbB3*^-/- ^TEB from a 7-week post-transplantation gland. cc3, cleaved caspase 3; H&E, hematoxylin and eosin; IgG, negative control antibody; E-cad, E-cadherin; P-cad, P-cadherin; SMA, smooth muscle actin; TEB, terminal end bud.

A cross-section of a classical TEB reveals two distinct epithelial cell layers. The outer layer of cap cells at the advancing end of the buds merges into the more distal myoepithelial layer [30,31]. Cap cells are active sites for proliferation in the TEB and the progenitors for the myoepithelial cells that line the duct and the interior body and luminal cells. The second layer encompasses the inner, multilayered body cells that line the lumen and are thought to be major sites of apoptosis in TEBs [32,33]. Both of these cell types are apparent in *ErbB3*^+/+ ^TEBs at 4 weeks after transplantation (Figure 4a). The single layer of cap (and myoepithelial) cells is marked by expression of SMA and the adhesion molecule P-cadherin (Figure 4b,d), whereas multilayered body cells express the adhesion molecule E-cadherin (Figure 4c) [34,35]. There is a large lumen surrounded by the body cells in each of the consecutive stained sections from the *ErbB3*^+/+ ^gland. The *ErbB3*^-/- ^TEBs have the same general organization, but they differ in the type and severity of the defect (Figure 4, bottom four rows). The first *ErbB3*^-/- ^TEB (Figure 4e–i) has a single layer of cap cells (Figure 4h) and a multilayered body cell compartment (Figure 4g); however, there are large vacuolated spaces between and the cap and body cell layers. The second *ErbB3*^-/- ^TEB (Figure 4j–n) also has some spaces between the cap and body cell layers, and the lumen is filled with cells. There is an influx of cells that express smooth muscle actin in the body cell compartment (Figure 4l) and do not express E-cadherin like the surrounding body cells (figure 4m). In a more severely defective *ErbB3*^-/- ^TEB (Figure 4o–s), there are fewer body cells. There is a single layer of cap cells surrounding a large space (Figure 4q), and few body cells (Figure 4p) that surround a small luminal space. Although the three 4-week TEBs in Figure 4 are from different mice, each of these three types of *ErbB3*^-/- ^TEB defects can be found in the same gland and in glands from different mice. The 7-week *ErbB3*^-/- ^TEB (Figure 4t–w) is disorganized in that there is a multilayered SMA-positive cap/myoepithelial cell compartment, with large spaces with the layers (Figure 4u). The normally multilayered body cells are a single layer surrounding a small lumen (Figure 4v).

**Figure 5:**
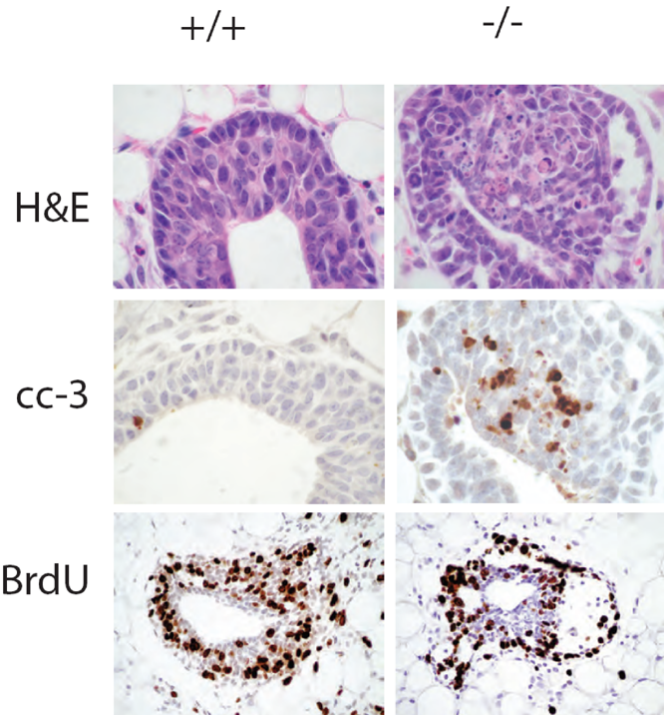
Apoptosis in TEBs. H&E stained TEBs in +/+ transplanted glands (top left) have few apoptotic nuclei and one cleaved caspase 3 positive (brown stain) cell (middle left). H&E stained TEBs in -/- transplanted glands (top right) have numerous apoptotic nuclei and cleaved caspase 3 positive cells (middle right). BrdU-positive cells (brown stain) are present in both the +/+ transplanted glands (bottom left) and in the -/- transplanted glands (bottom right). BrdU, 5-bromo-2-deoxyuridine; cc3, cleaved caspase 3; H&E, hematoxylin and eosin; TEB, terminal end bud.

The TEB is the major site of proliferation and apoptosis in the developing mammary gland. The proximal cap cells are actively proliferating cells, whereas the body cells that line the duct undergo apoptosis to clear the lumen during growth of the duct [32,33]. A decrease in proliferation or an increase in apoptosis could result in the ductal penetration defect associated with *ErbB3*^-/- ^transplants. Histological analysis of the H&E stained *ErbB3*^-/- ^TEBs revealed fragmented condensed nuclei suggestive of apoptosis (Figure 5, top right) in comparison with *ErbB3*^+/+ ^TEBs (Figure 5, top left). Hence, we enumerated apoptotic bodies in fields of H&E sections from *ErbB3*^+ ^and *ErbB3*^-/- ^mammary glands. Of the seven null glands analyzed, the mean number of apoptotic bodies was 2.3 (standard deviation [SD] 2.0). Six wild-type or heterozygous glands had a mean of 0.8 apoptotic bodies (SD 0.5). The counts were also compared after normalization to the total number of epithelial nuclei in each sample. Among all glands analyzed, *ErbB3*^-/- ^TEBs (seven glands) had a mean ratio of apoptotic bodies to nuclei of 0.03 (SD 0.03), whereas *ErbB3*^+ ^glands (six glands) had a mean ratio of 0.01 (SD 0.01). In the four pairs only, *ErbB3*^-/- ^TEBs had a mean ratio of apoptotic cells to nuclei of 0.02 (SD 0.01), whereas *ErbB3*^+ ^glands had a mean ratio of 0.01 (SD 0.01).

The area of the *ErbB3*^-/- ^TEB with the most apoptotic nuclei also displayed an increase in cleaved caspase 3 positive cells (Figure 5). On average, *ErbB3*^+/+ ^TEBs had 2.15% cleaved caspase 3 positive cells, with a range of 0.4% to 5.4% positive. *ErbB3*^-/- ^TEBs had an average of 6.3% cleaved caspase 3 positive cells, with a range of 1.6% to 18.5% positive. Overall, then, there was a trend for null TEBs to show more signs of apoptosis, but the differences may not rise to the level of statistical significance.

Proliferation was not affected by the loss of ErbB3. Immunohistological analysis of BrdU labeling yielded similar results for *ErbB3*^+ ^TEBs (29% BrdU positive) and *ErbB3*^-/- ^TEBs (32% BrdU positive; Figure 5, lower panels).

## Discussion

Deletion of *ErbB3 *in the epithelium of the mouse mammary gland resulted in reduced ductal outgrowth that was evident at puberty, and endured through adolescence, pregnancy, and early lactation, establishing that ErbB3 is absolutely required in the epithelium during pubescent mammary gland development. This phenotype is more severe than that induced by knockout of any of the three other ErbBs in mammary epithelium, underscoring the importance of ErbB3 in mammary development. The postnatal requirement of ErbB3 in the stroma was not addressed in this study. This ductal outgrowth/penetration phenotype varied in severity. Lobuloalveolar development was not affected in *ErbB3*-null glands. Lack of epithelial ErbB3 affected the phenotype of TEBs. TEBs were more numerous but smaller in size in the *ErbB3*^-/- ^outgrowths. Apoptotic cells were more common within the TEBs in the null glands, but there was no apparent change in the proliferative cells.

In related experiments, Qu and coworkers [36] inhibited ErbB3 expression in the mammary gland by using Cre recombinase to invert a transcriptional suppressor integrated in an intron. Mammary glands, analyzed at 4 weeks and 8 weeks, had lower ductal density, fewer branches, and fewer TEBs. TEB morphology was not discussed, and neither was development at later time points. The limited analysis, the fact that the experimental approach involved reduction, not elimination, of ErbB3 expression, and other technical differences make it difficult to compare that report with the present study directly.

The pubescent epithelial outgrowth/penetration defect we obtained was accompanied by an increase in the branch density in *ErbB3*-null glands. Mammary branching is modulated by diverse factors, including members of the EGFR family (for review, see [37]). The interaction between the ErbB family and matrix metalloproteinases (MMPs), in particular, is known to be important in branching. The EGFR promotes activation of MMP-2 and MMP-14, which are important for lung branching and morphogenesis [38]. In the mammary gland, MMP-2 is important for ductal elongation whereas MMP-3 promotes side branching [39,40]. Disruption of the MMP network by the lack of ErbB3 could result in decreased ability of the ducts to penetrate the surrounding stroma.

Despite the importance of branching, other abnormalities in the *ErbB3*-null glands are likely also to contribute to the outgrowth/penetration defect. TEBs are epithelial structures found at the end of a growing duct in the pubescent gland and are responsible for growth and penetration of the normal epithelial tree into the surrounding fat pad. TEBs are evident at the tips of the advancing ducts until the edge of the fat pad is reached, at which point they regress. In the *ErbB3*-null glands there was an increase in the number of TEBs, probably due to the increase in branching and consequently in the number of ducts, but a decrease in the overall size of the TEBs. On a histological level, the *ErbB3*^-/- ^TEBs had a range of structural abnormalities that could account for their small size (Figure 4). Some of the TEBs were relatively normal in organization, with an outer single layer of cap and myoepithelial cells marked by P-cadherin expression, an inner multilayered body cell compartment marked by E-cadherin expression, and a central cleared luminal space. A majority of the TEBs had some structural defect including large spaces between the cap/myoepithelial cell layer and the body cell layer, and/or filled in luminal spaces. The most severe TEB abnormalities were detected at 7 weeks after transplantation. The cap/myoepithelial cell compartment appeared to become multilayered and invaded or co-inhabited the body cell compartment, which itself was numerically diminished. This could occur through loss of normal compartmentalization boundaries, and even invasion of the inner layers by the outer layer, perhaps accompanying loss of body cells. Dysregulation of the P-cadherin or E-cadherin adhesion systems could account for some of these structural abnormalities, because they are important for maintaining the boundaries as is netrin/neogenin signaling. It is possible that changes in differentiation of progenitor cells alter the balance of these populations.

With the information available, the most likely source of the TEB aberrations is an increased rate of apoptosis. A change in the normal balance between proliferation and apoptosis in the TEBs of a pubertal mammary gland would affect the ability of the epithelial tree to fill the fat pad. The proliferative cap cells are typically located at the distal end of the TEB, whereas the apoptotic cells are located in the inner body cell layer surrounding the hollow lumen [32]. The pattern of apoptotic cells in the TEBs suggests that apoptosis is responsible for clearing of the lumen within the growing duct. In the *ErbB3*-null glands, proliferation was not affected, but apoptosis as detected by cleaved caspase 3 immunohistochemistry and the presence of apoptotic figures in H&E-stained sections was increased in comparison to *ErbB3*^+ ^glands. ErbB3 has six binding sites for the p85 adaptor subunit of PI3K and is strongly linked to prosurvival signaling through the PI3K/Akt pathway. The uncoupling of ErbB3 from the PI3K/Akt pathway in *ErbB3*-null outgrowths could result in the increase in apoptosis detected in the TEBs. An increase in apoptosis would explain the large number of cells in the normally cleared luminal space at 4 weeks after transplantation and then the apparent decrease in body cells numbers in TEBs at 7 weeks in ErbB3-null glands.

The *ErbB3*-null pubescent mammary gland phenotype might not be predicted based on earlier studies showing only limited expression of ErbB3 in the pubescent gland. However, implantation of the NRG1 in the pubescent mammary gland stimulated epithelial growth [23]. Hence, one of the NRG receptors ErbB3 and/or ErbB4 can function at this stage of mammary development. Because loss of ErbB3 function, but not loss of ErbB4 function, compromised pubescent mammary development, it is likely that ErbB3 was activated by the implanted ligand. However, ErbB3 is devoid of kinase activity and requires a heterodimerization partner for activation. Among the potential heterodimerization partners EGFR, ErbB2, and ErbB4, only EGFR and ErbB2 are required for ductal outgrowth in the pubescent mammary gland. EGFR is expressed in both the epithelium and stroma, but it is only required in the stroma during pubescent mouse mammary gland development [41]. Hence, ErbB2 may be the unique co-receptor, but EGFR and ErbB4 may work redundantly with ErbB3.

The epithelial null phenotype for ErbB2 [26] most closely resembles the phenotype for *ErbB3 *null, consistent with the importance of ErbB2/ErbB3 signaling at pubescence. *ErbB2*-null mammary buds transplanted into wild-type fat pads develop into ductal tree that grossly resembles the *ErbB3*-null phenotype. However, in contrast to *ErbB3 *nulls, the defect was present in the pubescent gland, but was not evident in adult glands or pregnancy. *ErbB2*-null TEBs also had structural defects in the TEBs, characterized by a decrease in body cell number, an increase in the presence of cap/myoepithelial-like cells in the prelumenal compartment, and the presence of large luminal spaces. The similar requirement for ErbB2 and ErbB3 in the epithelium of the pubescent mammary gland strongly implies that the ErbB2/ErbB3 heterodimer is important in the epithelium at this stage in development.

The heterodimerization partner(s) dictates the range of ligands capable of activating ErbB3 in the pubescent mammary gland. Because ErbB2 does not bind soluble ligands, the ErbB2/ErbB3 heterodimer would need to be activated by ligand-bound ErbB3. However, because growth factors in this family are activated by proteolysis, further work will be required to determine the importance of these agonists in pubescent mammary development. Knockout studies have shown that NRG1 isoform α is required, but in adult maturation of the mammary gland [28].

If ErbB3 is heterodimerized with EGFR, then other ligands could be responsible for transactivation of ErbB3. Of the known EGFR ligands, amphiregulin has been shown to be important and upregulated during pubescent mammary gland development [42,43]. With the mammary defects in amphiregulin-knockout mice, EGFR is probably signaling in the epithelium either as a homodimer or heterodimer with ErbB2 and/or ErbB3 in the pubescent mouse mammary gland.

ErbB family members are over-expressed because of amplification or have activating mutations in a variety of human cancers. Mutated *EGFR *has been found in a subset of NSCLCs, and *ERBB2 *is amplified and over-expressed in up to 30% of human breast cancers, which is associated with a poorer clinical outcome. In both contexts, the ErbBs are validated therapeutic targets. ErbB3 may be quite important in governing the signaling output of the activated ErbBs, initial response to therapies, and in the development of drug resistance. For example, some NSCLC patients with EGFR mutations with a good clinical response to EGFR inhibitors gefitinib or erlotinib initially, but later develop resistance. In one study, half of these patients developed a second activating mutation, whereas the other half had amplified *MET*, which encodes a receptor tyrosine kinase activated by hepatocyte growth factor [18] (for review, see [19]). The NSCLC tumors escaped tyrosine kinase inhibition because MET activates ErbB3 and the prosurvival PI3K kinase pathway. In human breast cancer cells that over-express ErbB2 and have also developed resistance to tyrosine kinase inhibitor therapy, there is active PI3K/Akt via ErbB3 signaling [15]. In summary, therapies that target ErbB tyrosine kinase activities, kinase inactive ErbB3 and the downstream PI3K/Akt signaling may evade inhibition. A better understanding of ErbB3 in normal mammary gland signaling will reveal the nature of endogenous regulators, and may be beneficial to development of future directed therapies.

## Conclusion

The *ErbB3*^-/- ^mammary outgrowth defect was associated with a decrease in the size of the TEBs, and increases in branch density, in the number of terminal end buds, and in the number of luminal spaces. Proliferation rates were not affected by the lack of *ErbB3*, but there was a trend toward increased apoptosis in *ErbB3*^-/- ^TEBs. Hence, endogenous ErbB3 regulates morphogenesis of mammary epithelium, most likely through impact on cell survival and other ErbB3-regulated processes.

## Abbreviations

BrdU: 5-bromo-2-deoxyuridine; EGFR: epidermal growth factor receptor; H&E: hematoxylin and eosin; IHC: immunohistochemistry; MMP: matrix metalloproteinase; NRG: neuregulin; NSCLC: non-small-cell lung carcinoma; PI3K: phosphatidylinositol 3'-kinase; SD: standard deviation; SMA: α-smooth muscle actin; TEB: terminal end bud.

## Competing interests

The authors declare that they have no competing interests.

## Authors' contributions

AJ-F, GB, and JB carried out the research. FT and CB conducted histological analysis. JKD provided statistical consult and figure design. AJ-F and DFS participated in the design of the study and drafted the manuscript. DFS conceived the study.

## Supplementary Material

Additional file 1 A pdf document showing expression of ErbB3. Immunoblot of empbryonic day (E)12.5 whole embryo lysates probed with anti-ErbB3 antibody (Santa Cruz Biotechnology SC285, 1:1,000) from wild type (+/+), heterozygote (+/-), and (-/-) embryos showed gene dose-dependent expression of ErbB3. Loading control is glyceraldehyde 3-phosphate dehydrogenase detected by immunoblotting with antibody SC25778. Click here for file
