# Supplementary figures and images for: ErbB3 is required for ductal morphogenesis in the mouse mammary gland
Source: Breast Cancer Res. 2008 Nov 18;10(6):R96. doi: 10.1186/bcr2198 (PMC2656891; doi:10.1186/bcr2198)

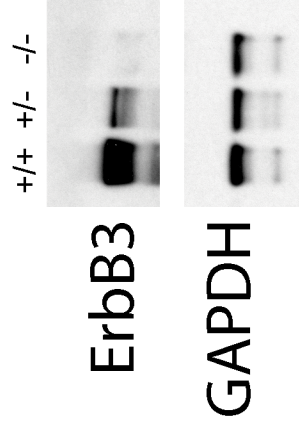

Supplement: Additional file 1 — A pdf document showing expression of ErbB3. Immunoblot of empbryonic day (E)12.5 whole embryo lysates probed with anti-ErbB3 antibody (Santa Cruz Biotechnology SC285, 1:1,000) from wild type (+/+), heterozygote (+/-), and (-/-) embryos showed gene dose-dependent expression of ErbB3. Loading control is glyceraldehyde 3-phosphate dehydrogenase detected by immunoblotting with antibody SC25778. [file bcr2198-S1.pdf]
